# Acute internal medicine physicians’ clinical intuition based on acute care telephone referral: A prospective study

**DOI:** 10.1371/journal.pone.0305566

**Published:** 2024-06-14

**Authors:** Paul M. E. L. van Dam, Roberto E. Lasso Peña, Jody A. Mommertz, Hella F. Borggreve, Nicole P. H. van Loon, Noortje Zelis, Dewa Westerman, Ronald M. A. Henry, Dirk Posthouwer, Jochen W. L. Cals, Patricia M. Stassen

**Affiliations:** 1 Department of Internal Medicine, Division of General Internal Medicine, Section Acute Medicine, Maastricht University Medical Center +, Maastricht, The Netherlands; 2 Department of Medical Microbiology, Maastricht University Medical Center+, Maastricht, The Netherlands; 3 Department of Family Medicine, Care and Public Health Research Institute (CAPHRI), Maastricht University, Maastricht, The Netherlands; 4 School for Cardiovascular Diseases (CARIM), Maastricht University, Maastricht, The Netherlands; Universiteit Antwerpen, BELGIUM

## Abstract

**Introduction:**

In the Netherlands, most emergency department (ED) patients are referred by a general practitioner (GP) or a hospital specialist. Early risk stratification during telephone referral could allow the physician to assess the severity of the patients’ illness in the prehospital setting. We aim to assess the discriminatory value of the acute internal medicine (AIM) physicians’ clinical intuition based on telephone referral of ED patients to predict short-term adverse outcomes, and to investigate on which information their predictions are based.

**Methods:**

In this prospective study, we included adult ED patients who were referred for internal medicine by a GP or a hospital specialist. Primary outcomes were hospital admission and triage category according to the Manchester Triage System (MTS). Secondary outcome was 31-day mortality. The discriminatory performance of the clinical intuition was assessed using an area under the receiver operating characteristics curve (AUC). To identify which information is important to predict adverse outcomes, we performed univariate regression analysis. Agreement between predicted and observed MTS triage category was assessed using intraclass and Spearman’s correlation.

**Results:**

We included 333 patients, of whom 172 (51.7%) were referred by a GP, 146 (43.8%) by a hospital specialist, and 12 (3.6%) by another health professional. The AIM physician’s clinical intuition showed good discriminatory performance regarding hospital admission (AUC 0.72, 95% CI: 0.66–0.78) and 31-day mortality (AUC 0.73, 95% CI: 0.64–0.81). Univariate regression analysis showed that age ≥65 years and a sense of alarm were significant predictors. The predicted and observed triage category were similar in 45.2%, but in 92.5% the prediction did not deviate by more than one category. Intraclass and Spearman’s correlation showed fair agreement between predicted and observed triage category (ICC 0.48, Spearman’s 0.29).

**Conclusion:**

Clinical intuition based on relevant information during a telephone referral can be used to accurately predict short-term outcomes, allowing for early risk stratification in the prehospital setting and managing ED patient flow more effectively.

## Introduction

Assessing the severity of the patient’s illness is an important challenge for physicians in the emergency department (ED). This risk stratification influences the urgency of diagnostic testing, treatment, and clinical decision-making. In the Netherlands, nearly all ED patients are referred after initial triage by a health professional (i.e. general practitioner (GP) or hospital specialist). The GP provides primary care to low-risk patients, and high-risk patients are referred to secondary care [1, 2]. High-risk patients (i.e. who are in need of (acute) hospital care) have to receive treatment in a timely fashion, as delays lead to overcrowding in the ED and higher mortality rates [3–6]. Rapid and accurate discrimination between high and low-risk patients is therefore an important aspect of emergency care [7].

Clinical intuition is a non-analytic, subconscious and instinctive process, through which physicians assess the severity of the patient’s illness [8–11]. The ED physician’s clinical intuition has demonstrated commendable discriminatory performance in previous studies, predicting adverse outcomes in ED patients (i.e. hospital admission or short-term mortality) with an area under the receiver operating characteristics curve (AUC) ranging from 0.74 to 0.85 [12–15]. Therefore, clinical intuition is an important tool for discrimination between high and low-risk patients in the ED.

Clinical intuition based on information shared between the referring physician and the ED physician could offer a rapid and early risk stratification tool before the patient arrives at the ED. During the referral of a patient by telephone, the ED physician can potentially estimate the severity of the patient’s illness and the need for hospital admission in the prehospital setting. This early risk stratification would allow the ED physician to manage the patient flow in the ED more effectively. However, as far as we know, no studies have investigated the discriminatory performance of clinical intuition based on telephone referral information to predict adverse outcomes.

Therefore, in this prospective study, we aimed to investigate the discriminatory performance of the ED physician’s clinical intuition based on telephone referral information to predict short-term outcomes. In addition, we aimed to investigate on which information the ED physicians based their predictions.

## Methods

### Study design and setting

This prospective cohort study was performed at the ED of the Maastricht University Medical Center + (MUMC+). This is a combined secondary/tertiary care center in the Netherlands, with 22,000 ED visits every year. The medical ethics committee of the MUMC+ approved this study (METC 2018–0838) and waived the requirement of informed consent. This study was conducted and reported in accordance with the STROBE guidelines (Strengthening the Reporting of Observation studies in Epidemiology) (S1 Table) [16].

In contrast to many other countries where patients can visit the ED without referral by a health professional (open access ED), most non-trauma ED patients in the Netherlands are referred by a GP or hospital specialist. After this initial evaluation, the referring physicians call the ED physicians to inform them about the reasons for referral. Self-referral is possible, however higher costs have been introduced to discourage self-referral [2].

### Study sample

Adult patients (18 years or older) who were referred by telephone to the ED of the MUMC+ for assessment and treatment by an internist, were eligible for inclusion. In our ED, internists and their residents attend all patients with problems related to internal medicine and gastroenterology, in addition to non-differentiated non-trauma patients. Patients were included in the period between March 18^th^ and June 30^th^ 2022.

Patients were included by the on-call acute internal medicine (AIM) physician. AIM physicians are internists who are specialized in stabilization, diagnosis and treatment of patients with acute presentations of internal diseases in the ED, the medical ward and the outpatients clinic. Emergency physicians (EPs) are specialized in emergency medicine (medical patients as well as trauma patients) in the ED. In many Dutch hospitals, AIM physicians and EPs work together in the ED, making use of each other’s specific expertise [2]. The AIM physicians who participated in our study are specialists or residents in the last stage of their specialist training. Since the AIM physicians were predominantly present on weekdays during day shifts (and to a lesser extent in the evenings and weekends), the study sample was derived on a convenience sample.

### Questionnaire

Patients were included in the study using a questionnaire filled out by the AIM physician during or immediately after the telephone referral. The questions were answered before the patient arrived at the ED and the responses could not be changed afterwards. The questionnaire was developed based on questionnaires from previous studies [12, 14, 17–25], and adapted to be feasible in clinical practice in the ED. The questionnaire is shown in the supporting information (S1 Fig).

The questionnaire consisted of nine questions (Q): Q1) Does the referring physician know the patient? (Yes/No); Q2) Which information was given during the telephone referral? (Previous medical history, Alarm symptoms, Physical examination, Diagnostic testing, Previous treatment, Diagnosis); Q3) What is your gut feeling? (Everything fits/Sense of alarm) [26]; Q4) How severely ill is this patient? (0–100%); Q5) Which Manchester Triage System (MTS) urgency do you expect this patient to receive? (Red/Orange/Yellow/Green/Blue) [27]; Q6) What is the chance that this patient will be admitted to the hospital? (0–100%); Q7) To what department will this patient be admitted? (“General ward” or “Intensive care unit/Medium care unit (ICU/MCU)”; Q8) What is the chance that this patient will be admitted for longer than 7 days? (0–100%); Q9) What is the chance that this patient dies within 31 days? (0–100%).

### Data collection

From the ED charts and the electronic medical records, data were collected on age, sex, comorbidity based on the Charlson Comorbidity Index (CCI), mode of transportation to the ED, and triage category based on the MTS [27, 28]. The triage was performed by the triage nurse, who was unaware of the triage category predicted by the AIM physician. It was also recorded whether the referring physician knew the patient (i.e. was the referral based on a continuous clinical relationship or on the first clinical encounter?), and whether the AIM physician who received the telephone referral was the same as the physician who later made the treatment plan in the ED. The main reason for referral to the ED was recorded according to the International Classification of Diseases (ICD)-10 system [29]. The patients’ vital signs and the results of laboratory tests during the ED visit were also retrieved. Finally, data were collected on hospital admission, length of hospital stay, admission to ICU/MCU, and 31-day mortality. Data on mortality were verified using the medical records. In the Netherlands, all deaths are registered by the municipal administration office, and these data are linked to the medical records. Data collection was performed by medical students and resident doctors, who were not blinded for the results of the questionnaires. The quality of the data was checked by sample by two researchers, and discrepancies were resolved through discussion with another investigator.

### Outcomes

The primary outcomes for assessing the discriminatory performance of the AIM physician’s clinical intuition during telephone referral were hospital admission and MTS triage category in the ED. The secondary outcome was 31-day mortality.

### Statistical analysis

Regarding sample size, in order to investigate the discriminatory performance of clinical intuition in a prehospital setting to predict hospital admission, we aimed to comply with the rule of thumb to include approximately 100 patients who met the primary outcome, similar to other studies [30]. Assuming an admission rate of 65%, we calculated a required sample size of approximately 154 patients (65% of 154 patients equals 100 admitted patients). In order to address any selection bias in our convenience sample, we used the Chi square test to compare the age, hospital admission rate and MTS triage categories of our study sample to all ED patients with problems related to internal medicine or gastroenterology during the entire year of 2022.

Baseline characteristics were analyzed using descriptive statistics. Continuous variables were reported as medians with interquartile ranges (IQR), remaining variables were reported as totals with percentages. In case of missing estimates on clinical intuition or outcome, the patient could not be included in further analysis. To identify which information during the telephone referral is most important to predict the patients’ outcome, we performed univariate logistic regression analyses, and odds ratios (OR) with 95% confidence intervals (95% CI) were reported. For the purpose of the univariate logistic regression analysis, we divided the observed MTS triage category into urgent (i.e. yellow, orange and red) and non-urgent (i.e. blue and green). A p-value < 0.05 was considered to be statistically significant. The agreement between the predicted and observed MTS triage category was analyzed using the Spearman’s rank correlation and the intraclass correlation coefficient (ICC). The discriminatory performance of clinical intuition was assessed by calculating an AUC with 95% CI. An AUC of 0.5 corresponds with very poor discriminatory performance, whereas an AUC of 1.0 means perfect accuracy. Calibration was assessed by visually inspecting the calibration plot.

All data were analyzed using IBM SPSS Statistics for Windows, IBM Corporation, Armonk NY, version 25.0. DeLong tests were performed in R, version 4.0.0.

## Results

### Study sample

During the study period, 333 ED patients were included after telephone referral (Table 1). The median age was 70 years (IQR 56–80), and 191 patients (57.4%) were male. The median time between referral and arrival to the ED was 54 minutes (IQR: 31–97 minutes).

**Table 1:** Patient characteristics of the study sample.

	Study sample (n = 333)
Age, median (IQR), years	70 (56–80)
Male, n%	191 (57.4)
CCI, median (IQR)	2 (3–4)
Transport by ambulance, n%	95 (28.5)
Time to arrival (minutes), median (IQR)	54 (31–97)
Triage category (MTS), n%	
Blue (Non urgent)	1 (0.3)
Green (Standard)	142 (42.6)
Yellow (Urgent)	146 (43.8)
Orange (Very urgent)	38 (11.4)
Red (Immediate)	4 (1.2)
Main reason for referral to the ED ^a^, n%	
Gastrointestinal disease	60 (18.0)
Infectious disease	50 (15.0)
Malignancy	39 (11.7)
Cardiovascular disease	31 (9.3)
Respiratory disease	27 (8.1)
Urogenital disease	27 (8.1)
Hematologic disease	25 (7.8)
Endocrine or metabolic disease	17 (5.1)
Other	58 (17.4)
Outcomes	
Admission to hospital, n%	202 (60.7)
Length of hospital stay, median (IQR), days	5 (2–9)
Prolonged admission to hospital (>7 days), n%	64 (19.2)
ICU/MCU admission, n%	9 (2.7)
31-day mortality, n%	30 (9.0)

CCI, Charlson Comorbidity Index; ICU, intensive care unit; IQR, interquartile range; MCU, medium care unit; MTS, Manchester triage system

^a^ Recorded according to the International Classification of Diseases (ICD)-10 system

In total, 202 patients (60.7%) were admitted to the hospital, 188 patients (56.4%) were triaged as urgent (yellow, orange or red, according to MTS), and 30 patients (9.0%) died within 31 days after the ED visit. The 31-day follow-up period was complete for all patients.

During the study period, a total of 1664 patients visited our ED. We found no significant differences in MTS triage category and hospital admission rate of our study sample when compared to all ED patients with problems related to internal medicine or gastroenterology in 2022 (S2 Table).

### Questionnaires

In total, 10 AIM physicians with a median experience level of 17 years (IQR 13–22) participated in the inclusion of patients. The answers to the questions in the questionnaire are summarized in Table 2. In total, 172 patients (51.7%) were referred by a GP, 146 patients (43.8%) by a hospital specialist and 12 patients (3.6%) by another health professional (e.g. nursing home physician or ambulance). In most cases (63.0%), the referral was based on a continuous clinical relationship (i.e. the referring physician knew the patient). In 130 patients (39.0%), the AIM physician who received the telephone referral was the same as the physician who later made the treatment plan in the ED (S3 Table).

**Table 2:** Summary of the data from the questionnaires.

	Total sample (n = 333)	Referred by GP (n = 172)	Referred by specialist (n = 146)
Referring physician, n% ^a^			
General physician	172 (51.7)		
Hospital specialist	146 (43.8)		
Other (i.e. nursing home, ambulance)	12 (3.6)		
Referral based on continuous clinical relationship, n%	210 (63.0)	116 (67.4)	90 (61.6)
Information given during telephone referral, n%			
Medical history	275 (82.6)	137 (79.7)	126 (86.3)
Alarm symptoms	205 (61.6)	110 (64.0)	88 (60.3)
Physical examination	160 (48.0)	114 (66.3)	40 (27.4)
Additional diagnostic tests	97 (29.1)	66 (38.4)	31 (21.4)
Previous treatment	66 (19.8)	26 (15.1)	39 (26.7)
Preliminary diagnosis	181 (54.4)	95 (55.2)	77 (52.7)
Total number of items, median (IQR)	3 (2–4)	3 (2–4)	3 (2–4)
Clinical intuition			
Gut feeling (sense of alarm), n%	184 (55.3)	96 (55.8)	80 (54.8)
Severity of illness (0–100), median (IQR)	40 (30–60)	50 (30–60)	40 (30–60)
Predicted triage category (MTS)			
Blue (Non urgent)	15 (4.5)	10 (5.8)	5 (3.4)
Green (Standard)	120 (36.0)	59 (34.3)	56 (38.4)
Yellow (Urgent)	155 (46.5)	73 (42.4)	73 (50.0)
Orange (Very urgent)	42 (12.6)	29 (16.9)	12 (8.2)
Red (Immediate)	0	0	0
Predicted outcome			
Chance of admission to hospital, median (IQR)	80 (60–100)	80 (60–100)	80 (50–95)
Predicted admission to ICU/MCU, n%	4 (1.2)	1 (0.6)	2 (1.4)
Chance of prolonged admission (>7 days), median (IQR)	30 (5–50)	30 (0–50)	27.5 (10–50)
Chance of 31-day mortality, median (IQR)	10 (5–20)	10 (5–25)	10 (3–20)
Observed outcomes, n%			
Admission to hospital	202 (60.7)	107 (62.2)	86 (58.9)
ICU/MCU admission	9 (2.7)	4 (2.3)	5 (3.4)
31-day mortality	30 (9.0)	20 (11.6)	9 (6.2)

ICU, intensive care unit; IQR, interquartile range; MCU, medium care unit; MTS, Manchester triage system.

^a^ In 3 patients (0.8%) the referring physician was not recorded.

The information given during telephone referral was recorded (Table 2). Out of the 6 items that the AIM physician could fill out on the questionnaire, a median of 3 items (IQR 2–4) was scored. This number of items was comparable in those who were referred by a GP and those who were referred by a specialist. The medical history was discussed most frequently (82.6%), followed by the presence of alarm symptoms (61.6%) and the results of physical examination (48.0%).

### Prediction of hospital admission

In our sample, 202 patients (60.7%) were admitted to the hospital and 9 patients (2.7%) were admitted to ICU (Table 1). The median length of hospital stay was 5 days (IQR 2–9) and 64 patients (19.2%) were admitted longer than 7 days. The AIM physicians predicted a median chance of hospital admission of 80% (IQR: 60–100) and a median chance of prolonged hospital admission (>7 days) of 30% (IQR: 5–50). The predicted number of ICU/MCU admissions was 4 (1.2%), and 3 out of the 9 ICU/MCU admissions were predicted correctly.

**Fig 1:**
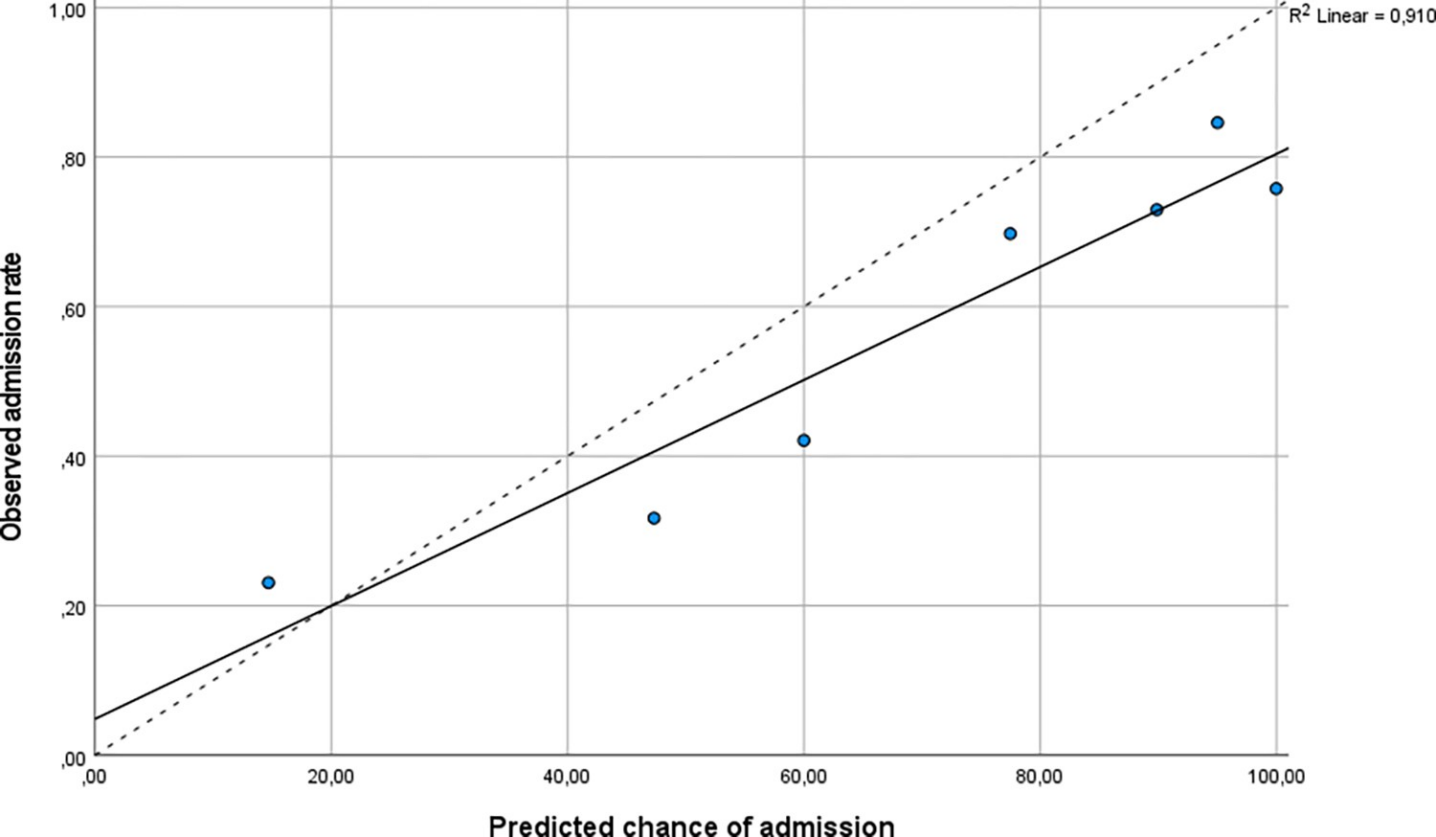
Calibration plot of the predicted chance of hospital admission. Calibration plot of the predicted chance of hospital admission (x axis) using clinical intuition. The calibration plot shows average overestimation of the chance of admission and a slope of < 1 compared to the dotted line (which would indicate perfect calibration).

The clinical intuition of the AIM physicians showed good discriminatory performance regarding hospital admission with an AUC of 0.72 (95% CI: 0.66–0.78). The calibration plot showed average overestimation of the chance of admission by the AIM physician and a slope of <1 (Fig 1). In a subgroup analysis of patients where the AIM physician who received the telephone referral was the same as the physician who later made the treatment plan in the ED, we found no significant differences in observed outcomes or discriminatory performance regarding hospital admission (S3 Table).

Of the patients who were referred by a GP, 107 patients (62.2%) were admitted to the hospital. The clinical intuition yielded an AUC of 0.74 (95% CI: 0.65–0.82) regarding admission. Of the patients who were referred by a hospital specialist, 86 patients (58.9%) were admitted and the clinical intuition yielded an AUC of 0.67 (95% CI: 0.58–0.77).

The univariate logistic regression analysis showed that age 65 years or older and the presence of a sense of alarm in the AIM physician were the significant predictors of hospital admission during telephone referral (Table 3).

**Table 3:** Univariate logistic regression analysis for hospital admission and 31-day mortality.

Predictor	Hospital admission		31-day mortality	
	Odds ratio (95% CI)	P-value	Odds ratio (95% CI)	P-value
Age ≥65 years	1.99 (1.20–3.31)	0.008	2.08 (0.80–5.46)	0.135
Referral by GP	1.15 (0.70–1.89)	0.590	1.59 (0.67–3.76)	0.294
Referral based on continuous clinical relationship	1.00 (0.59–1.70)	0.996	0.64 (0.27–1.48)	0.291
3 or more items provided during referral	1.57 (0.84–2.96)	0.160	1.17 (0.40–3.47)	0.773
4 or more items provided during referral	0.62 (0.32–1.22)	0.168	1.25 (0.44–3.56)	0.674
5 or more items provided during referral	0.68 (0.29–1.61)	0.386	0.82 (0.20–3.30)	0.780
AIM physician has sense of alarm	1.89 (1.16–3.08)	0.011	1.97 (0.83–4.70)	0.126

CI, confidence interval; GP, general practitioner.

### Prediction of MTS triage category

The majority of patients (56.4%) were triaged at the ED by the triage nurse as urgent (yellow, orange or red, according to MTS) and most patients were triaged as green or yellow (Table 4). According to the AIM physicians, green (120 patients, 36.0%) and yellow (155 patients, 46.5%) were the most frequently predicted triage category as well.

**Table 4:** Predicted versus observed MTS triage category in total study sample.

	Observed triage category						
**Predicted triage category**		Blue	Green	Yellow	Orange	Red	**Total predicted**
	Blue	0	10	5	0	0	15
	Green	1	63	48	7	0	120
	Yellow	0	59	75	19	1	155
	Orange	0	9	18	12	3	42
	Red	0	0	0	0	0	0
	**Total observed**	1	142	146	38	4	

MTS, Manchester Triage System.

Green fields represent agreement between predicted and observed triage categories. Yellow fields represent deviation of the prediction by one triage category. Red fields represent deviation of the prediction by more than one triage category.

In total, the predicted triage category by the AIM physician was similar to the triage category observed in the ED in 45.2%, and did not deviate by more than one urgency level in 92.5% of the patients (Table 4). The ICC was 0.48 (95% CI: 0.35–0.58, p <0.001) and Spearman’s correlation coefficient was 0.29 (95% CI: 0.19–0.39, p <0.001), indicating moderate to fair agreement between the predicted and observed MTS triage category. The accuracy of the predicted triage category did not depend on whether the referring physician knew the patient as in both the correct and incorrect predictions, the physician knew the patient in approximately 70% of cases (70.1% and 68.2%). The predicted MTS triage category by the AIM physician showed poor discriminatory performance regarding hospital admission with an AUC of 0.61 (95% CI: 0.55–0.67).

We found that in both patients who were referred by a GP and those who were referred by a hospital specialist, there was at most a moderate correlation between the predicted and observed triage category. In patients who were referred by a GP, the ICC was 0.52 (CI 95%: 0.35–0.65, p <0.001) and Spearman’s correlation coefficient was 0.33 (95% CI: 0.19–0.47, p<0.001), indicating fair agreement (S4 Table). In the patients who were referred by a hospital specialist, the ICC was 0.36 (CI 95%: 0.11–0.54, p = 0.004) and the Spearman’s correlation coefficient was 0.20 (95% CI: 0.04–0.36, p = 0.014), indicating poor agreement (S5 Table).

The univariate logistic regression analysis showed that the presence of a sense of alarm in the AIM physician was the only significant predictor of both the predicted and observed MTS triage category during telephone referral (Table 5).

**Table 5:** Univariate logistic regression analysis for MTS triage category.

Predictor	Predicted MTS triage category ^a^		Observed MTS triage category ^a^	
	Odds ratio (95% CI)	P-value	Odds ratio (95% CI)	P-value
Age ≥65 years	0.97 (0.57–1.65)	0.899	0.66 (0.40–1.10	0.109
Referral by GP	1.09 (0.65–1.82)	0.757	0.86 (0.53–1.41)	0.553
Referral based on continuous clinical relationship	0.94 (0.54–1.64)	0.837	1.30 (0.77–2.20)	0.321
3 or more items provided during referral	0.97 (0.51–1.82)	0.912	0.84 (0.46–1.53)	0.564
4 or more items provided during referral	0.87 (0.44–1.72)	0.680	1.06 (0.55–2.02)	0.866
5 or more items provided during referral	1.80 (0.70–4.62)	0.223	0.95 (0.40–2.24)	0.901
AIM physician has sense of alarm	4.66 (2.81–7.73)	<0.001	1.82 (1.13–2.93)	0.014

CI, confidence interval; GP, general practitioner; MTS, Manchester triage system.

^a^ Analysis was performed as logistic regression analysis, dividing MTS triage categories into urgent (yellow, orange, and red) and non-urgent (blue and green).

### Prediction of mortality

**Fig 2:**
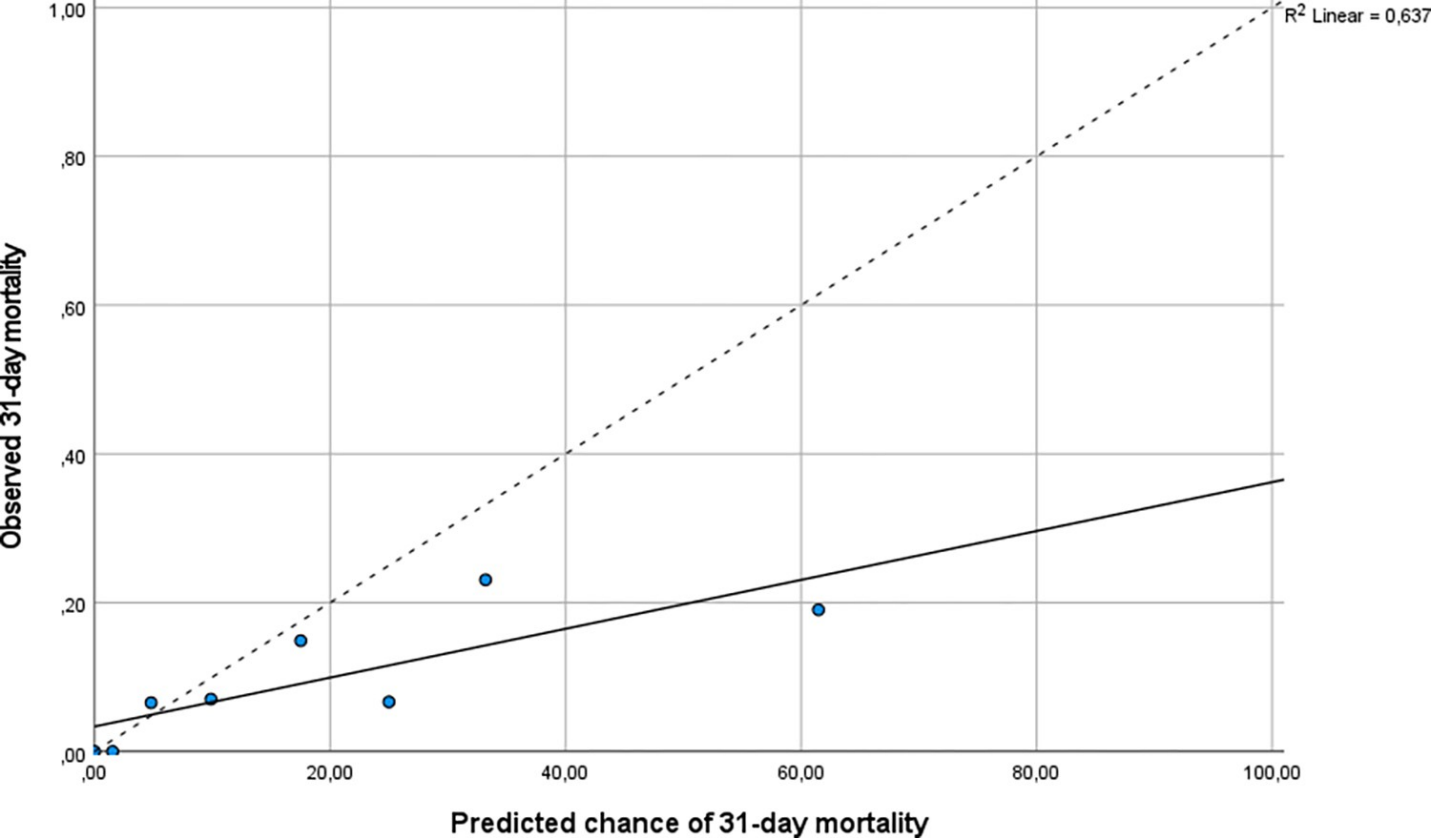
Calibration plot of the predicted chance of 31-day mortality. Calibration plot of the predicted chance of 31-day mortality (x axis) using clinical intuition. The calibration plot shows average overestimation of the chance of mortality and a slope of < 1 compared to the dotted line (which would indicate perfect calibration).

In our sample, 30 patients (9.0%) died within 31 days after the ED visit (Table 1). The AIM physicians predicted a median chance of 31-day mortality of 10% (Table 2). The clinical intuition of the AIM physicians showed good discriminatory performance to predict 31-day mortality with an AUC of 0.73 (95% CI: 0.64–0.81). The calibration plot showed average overestimation of the 31-day mortality risk by AIM physicians and a slope of <1 (Fig 2).

The univariate logistic regression analysis showed that age 65 years or older and the presence of a sense of alarm in the AIM physician were the significant predictors of 31-day mortality (Table 3). However, the ORs were not statistically significant.

## Discussion

In this prospective study, we investigated the discriminatory performance of the clinical intuition of AIM physicians based on telephone referral of ED patients to predict short-term outcomes. In our cohort of 333 patients, the AIM physicians filled out questionnaires during or immediately after telephone referral by a GP, specialist or other health professional. The AIM physicians’ clinical intuition to predict hospital admission showed good discriminatory performance with an AUC of 0.72, and the calibration plot showed average overestimation of the chance of admission. The discriminatory performance to predict hospital admission was slightly higher in patients who were referred by a GP than in those who were referred by a hospital specialist (AUC 0.74 versus 0.67). We found that age 65 years or older and the presence of a sense of alarm in the AIM physician were significant predictors of hospital admission during telephone referral. The AIM physicians predicted the same MTS triage category in the ED in just under half of the cases (45.2%), but in the majority of the cases (92.5%) the prediction did not deviate by more than one urgency level. The accuracy of this prediction did not depend on who referred the patient (GP or hospital specialist), nor on whether the referring physician knew the patient. We found that the presence of a sense of alarm in the AIM physician was the only significant predictor of the MTS triage category. The AIM physicians’ clinical intuition to predict 31-day mortality showed good discriminatory performance with an AUC of 0.73. However, given the low number of deaths, our study was underpowered to adequately analyze this secondary outcome.

### Clinical intuition in the prehospital setting

Our findings show that overall the discriminatory performance of the clinical intuition of AIM physicians during telephone referral of ED patients is good. Therefore, AIM physicians have the opportunity to assess the severity of the patients’ illness in the prehospital setting. In our cohort, the patients took a median of 54 minutes to arrive at the ED after telephone referral. Therefore, the prediction in the prehospital setting has a longitudinal character. This may affect the accuracy of the prediction, since the patients’ clinical condition can improve or worsen between referral and arrival. The accuracy of the prediction could also be influenced by who the referring physician is. GPs usually refer after clinically assessing the patient themselves. When a hospital specialist refers a patient, this assessment usually takes place by telephone. However, in our cohort we found no significant differences in discriminatory performance between these groups. Furthermore, whether the referring physician knew the patient did not play a major role in the accuracy of the prediction.

Our findings regarding the discriminatory performance of clinical intuition are in line with those of other studies that report discriminatory performance of clinical intuition regarding short-term mortality or hospital admission with an AUC ranging from 0.71 to 0.77 [12–14, 31]. However, these studies were all performed in an hospital setting (i.e. ED or acute medical unit). To our knowledge, this is the first prospective study to investigate the discriminatory performance of clinical intuition based on telephone referral in a prehospital phase. One study in the UK assessed the accuracy of telephone triage in identifying the need for emergency care in more than 40,000 patients with suspected COVID-19 infection [32]. The authors found that 60% of the patients received a non-urgent assessment and were indeed at low risk of adverse outcome. Another study assessed the accuracy to identify life-threatening conditions during telephone calls concerning patients with loss of consciousness in a Danish police-operated emergency call-center [33]. They found a sensitivity of 82% and a positive predictive value of 39%.

In our study, the AIM physician was able to make a good estimate of the risk of adverse outcome based on a telephone referral of a patient to the ED. The calibration plot showed average overestimation of the chance of admission and mortality, indicating that the physician uses a safe margin when estimating the severity of the patients’ illness. In our study, the accuracy to predict the MTS triage category was lower and there was only fair agreement between the predicted and observed triage category. Previous studies showed inferior performance of the MTS triage category in older medical patients [34]. In our study, the MTS showed poor discriminatory performance to predict hospital admission and with an AUC of only 0.61.

Our group of AIM physicians consisted of ten physicians with a median of 17 years (IQR: 13–22) of experience. The results of our study show that our group of experienced physicians can play an important coordinating role within the acute care chain, creating a possibility for early risk stratification, to assist in the logistics of a crowded ED and to guide allocating healthcare resources. Our results also emphasize the importance of providing information by the referring GP or hospital specialist.

### Study limitations

Our study has several limitations. First, our study was performed in a single medical centre, limiting the generalizability of the results. However, our sample of ED patients and the number of participating AIM physicians was relatively large, and the follow-up of all patients was complete. Second, there is a risk of bias since we used a convenience sample. To address selection bias, we compared the age, MTS triage categories and hospital admission rate of our study sample with all medical (non-trauma) ED patients during an entire year and in a previous study in our ED, and found no differences (S2 Table) [35]. Therefore, the study sample appears to be a representative sample of the ED population at the MUMC+. Third, there is a risk of bias since in 130 patients (39.0%) the AIM physician who received the telephone referral was the same as the physician who later made the treatment plan in the ED. However, in a subgroup analysis in these patients we found no significant differences in observed patient outcomes or discriminatory performance (S3 Table). Last, in studies assessing clinical intuition there is a theoretical risk of bias caused by the Hawthorne effect, because completing questions regarding clinical intuition may influence the outcome of the patients’ treatment in the ED (e.g. decisions regarding hospital admission). In our study, the questionnaires were completed in the prehospital phase and were not modified afterwards. In addition, the physician who treated the patient in the ED was often not the one who received the telephone referral, as the median time between the referral and the patients’ arrival in the ED was 54 minutes.

## Conclusion

In conclusion, the clinical intuition of an AIM physician based on a telephone referral can be used to accurately predict hospital admission and the triage category before the patients enter the ED. The results of our study suggest that when experienced physicians fulfil a coordinating role within the acute care chain, this creates a possibility for early risk stratification, to assist in the logistics in a crowded ED and to guide allocating healthcare resources.

## Supporting information

S1 Fig Questionnaire used in this study. (DOCX)

S1 Table STROBE statement, checklist of items that should be included in reports of observational studies. (DOCX)

S2 Table Patient characteristics of the study sample. (DOCX)

S3 Table Subgroup analysis in patients where the AIM physician who received the telephone referral was the same as the physician who made the treatment plan in the ED. (DOCX)

S4 Table Predicted versus observed MTS triage category in patients referred by the GP. (DOCX)

S5 Table Predicted versus observed MTS triage category in patients referred by hospital specialists. (DOCX)
