# Supplementary material for: Acute internal medicine physicians’ clinical intuition based on acute care telephone referral: A prospective study
Source: PLoS One. 2024 Jun 14;19(6):e0305566. doi: 10.1371/journal.pone.0305566 (PMC11178206; doi:10.1371/journal.pone.0305566)
Supplement: S1 Fig — (DOCX) [file pone.0305566.s001.docx]

**S2 Figure. Questionnaire used in this study.**

| Date: ……………………………... Phone call time: ……………… Initials AIM physician: …………..  Demographic information:  Sex: Male / Female  Patient name: ………………………………………….. Date of birth (dd-mm-jjjj): …………………………  Referring physician:  Name: …………………………………………..  Function: General practitioner / Specialist / other ………………………………  Knows the patient: Yes / No  Information given during telephone referral:  Medical history: Yes / No Additional diagnostic tests: Yes / No  Alarm symptoms: Yes / No Previous treatment: Yes / No  Physical examination: Yes / No Preliminary diagnosis: Yes / No  Preliminary diagnosis (GP / specialist): …………………………………………  Preliminary diagnosis (AIM physician): …………………………………………  Questionnaire:  What is your gut feeling: Sense of reassurance / Sense of alarm  How severely ill is this patient? (0- 100) ……………………………………………  Which Manchester Triage System (MTS) urgency do you expect this patient to receive?  □ Red (acute) - immediately  □ Orange (very urgent) - within 10 minutes  □ Yellow (urgent) - within 60 minutes  □ Green (standard) - within 120 minutes  □ Blue (not urgent) - within 240 minutes  What is the chance this patient will be admitted to the hospital? (0-100%) ………………….  To what department will this patient be admitted? General ward / ICU/MCU  What is the chance this patient will be admitted for longer than 7 days? (0-100%) …………………….  What is the chance this patient dies within 31 days? (0 to 100%) ……………………. |
| --- |
