# Supplementary material for: Acute internal medicine physicians’ clinical intuition based on acute care telephone referral: A prospective study
Source: PLoS One. 2024 Jun 14;19(6):e0305566. doi: 10.1371/journal.pone.0305566 (PMC11178206; doi:10.1371/journal.pone.0305566)
Supplement: S2 Table — (DOCX) [file pone.0305566.s003.docx]

**S3 Table. Patient characteristics of the study sample.**

|  | **Study sample (n = 333)** | **Annual total (n = 6816)** | **Chi square test**  **(p value) ^a^** |
| --- | --- | --- | --- |
| Age, median (IQR), years | 70 (56-80) | 66 (50-77) | 1.00 |
|  |  |  |  |
| Triage category (MTS), n% |  |  |  |
| Blue (Non urgent) | 1 (0.3) | 121 (1.8) | 0.91 |
| Green (Standard) | 142 (42.6) | 2479 (36.4) |  |
| Yellow (Urgent) | 146 (43.8) | 3193 (46.8) |  |
| Orange (Very urgent) | 38 (11.4) | 815 (12.0) |  |
| Red (Immediate) | 4 (1.2) | 74 (1.1) |  |
|  |  |  |  |
| Admission to hospital, n% | 202 (60.7) | 3894 (57.1) | 0.83 |

IQR, interquartile range; MTS, Manchester triage system
