# Supplementary material for: Acute internal medicine physicians’ clinical intuition based on acute care telephone referral: A prospective study
Source: PLoS One. 2024 Jun 14;19(6):e0305566. doi: 10.1371/journal.pone.0305566 (PMC11178206; doi:10.1371/journal.pone.0305566)
Supplement: S3 Table — (DOCX) [file pone.0305566.s004.docx]

**S4 Table. Subgroup analysis in patients where the AIM physician who received the telephone referral was the same as the physician who made the treatment plan in the ED.**

|  | **Same physician ^a^**  **(n = 130, 39.0%)** | **Not the same physician ^b^**  **(n = 203, 61.0%)** | **Total sample**  **(n = 333)** |
| --- | --- | --- | --- |
| Observed outcome |  |  |  |
| Admission to hospital, n% | 71 (54.6) | 131 (64.5) | 202 (60.7) |
| ICU/MCU admission, n% | 3 (2.3) | 6 (3.0) | 9 (2.7) |
| 31-day mortality, n% | 15 (11.5) | 15 (7.4) | 30 (9.0) |
|  |  |  |  |
| Discriminatory performance to predict admission, AUC (95% CI) | 0.72 (0.63-0.81) | 0.71 (0.64-0.79) | 0.72 (0.66-0.78) |

AUC, Area under the curve; CI, Confidence interval

^a^ Subgroup of patients where the AIM physician who received the telephone referral was the same as the physician who made the treatment plan in the ED.

^b^ Subgroup of patients where the AIM physician who received the telephone referral was **not** the same as the physician who made the treatment plan in the ED.
