# Supplementary material for: Acute internal medicine physicians’ clinical intuition based on acute care telephone referral: A prospective study
Source: PLoS One. 2024 Jun 14;19(6):e0305566. doi: 10.1371/journal.pone.0305566 (PMC11178206; doi:10.1371/journal.pone.0305566)
Supplement: S4 Table — (DOCX) [file pone.0305566.s005.docx]

**S5 Table. Predicted versus observed MTS triage category in patients referred by the GP.**

|  | **Observed triage category** | | | | | | |
| --- | --- | --- | --- | --- | --- | --- | --- |
| **Predicted triage category** |  | Blue | Green | Yellow | Orange | Red | **Total predicted** |
|  | Blue | 0 | 7 | 3 | 0 | 0 | 10 |
|  | Green | 0 | 32 | 22 | 5 | 0 | 59 |
|  | Yellow | 0 | 31 | 29 | 11 | 1 | 72 |
|  | Orange | 0 | 5 | 12 | 10 | 2 | 29 |
|  | Red | 0 | 0 | 0 | 0 | 0 | 0 |
|  | **Total observed** | 0 | 75 | 66 | 26 | 3 |  |

MTS, Manchester Triage System.

Green fields represent agreement between predicted and observed triage categories. Yellow fields represent deviation of the prediction by one triage category. Red fields represent deviation of the prediction by more than one triage category.
